# Dementia prevalence estimates in sub-Saharan Africa: comparison of two diagnostic criteria

**DOI:** 10.3402/gha.v6i0.19646

**Published:** 2013-04-03

**Authors:** Stella-Maria Paddick, Anna R. Longdon, Aloyce Kisoli, Catherine Dotchin, William K. Gray, Felicity Dewhurst, Paul Chaote, Raj Kalaria, Ahmed M. Jusabani, Richard Walker

**Affiliations:** 1 Institute of Neuroscience, Newcastle University, Newcastle upon Tyne, UK; 2 South Devon Healthcare NHS Foundation Trust, Torquay, UK; 3 Hai District Medical Centre, Boman'gombe, Kilimanjaro, Tanzania; 4 Northumbria Healthcare NHS Foundation Trust, North Tyneside General Hospital, North Shields, UK; 5 Institute for Ageing and Health, Newcastle University, Newcastle upon Tyne, UK; 6 Institute of Health and Society, Newcastle University, Newcastle upon Tyne, UK; 7 District Medical Office, Hai District Hospital, Boman'gombe, Hai, Tanzania; 8 Department of Radiology, Kilimanjaro Christian Medical Centre, Moshi, Kilimanjaro Region, Tanzania

**Keywords:** dementia, diagnosis, Tanzania, Africa

## Abstract

**Background:**

We have previously reported the prevalence of dementia in older adults living in the rural Hai district of Tanzania according to the *Diagnostic and Statistical Manual of Mental Disorders*, 4th edition (DSM-IV) criteria. The aim of this study was to compare prevalence rates using the DSM-IV criteria with those obtained using the 10/66 diagnostic criteria, which is specifically designed for use in low- and middle-income countries.

**Methods:**

In phase I, 1,198 people aged 70 and older were screened for dementia. A stratified sample of 296 was then clinically assessed for dementia according to the DSM-IV criteria. In addition, data were collected according to the protocol of the 10/66 Dementia Research Group, which allowed a separate diagnosis of dementia according to these criteria to be established.

**Results:**

The age-standardised prevalence of clinical DSM-IV dementia was 6.4% (95% confidence interval [CI] 4.9–7.9%) and of ‘10/66 dementia’ was 21.6% (95% CI 17.5–25.7%). Education was a significant predictor of ‘10/66 dementia’, but not of DSM-IV dementia.

**Conclusions:**

There are large discrepancies in dementia prevalence rates depending on which diagnostic system is used. In rural sub-Saharan Africa, it is not clear whether the association between education and dementia using the 10/66 criteria is a genuine effect or the result of an educational bias within the diagnostic instrument. Despite its possible flaws, the DSM-IV criteria represent an international standard for dementia diagnosis. The 10/66 diagnostic criteria may be more appropriate when identification of early and mild cognitive impairment is required.

Dementia and other non-communicable diseases are likely to become an increasing burden on health services in sub-Saharan Africa (SSA) as populations age and mortality and morbidity from communicable diseases, such as tuberculosis, malaria, and HIV/AIDS, begin to decline. Although dementia cannot be cured, early detection, allowing for effective management, can improve quality of life and can help reduce the burden of the disease on people with dementia, their carers, and healthcare providers (1).

Detection of dementia cases in SSA is hampered, among other reasons, by a lack of consensus on which diagnostic criteria are best suited to people living in SSA. This may explain, in part, why studies of dementia prevalence have reported such widely varying rates, for example, in urban Nigeria with prevalence rates in those aged 65 and older, of 2.3 and 10.1% (2, 3). The *Diagnostic and Statistical Manual of Mental Disorders*, 4th edition (DSM-IV), represents the current ‘gold standard’ for dementia diagnosis worldwide (4). However, it was developed in high-income countries, and its clinical utility in developing countries has been questioned due to its requirement that functional impairment must be present in order for a diagnosis to be made (5). In many areas of SSA, older people are not required to carry out the types of complex functional tasks that they would be if they lived in an urban environment in a high-income country. Moreover, older people in SSA often live within an extended family where younger members are available, and expected to assist elders in activities of daily living. Consequently, levels of cognitive decline which would be disabling to people in high-income countries may present much less of a burden to people living in SSA. Investigators from the 10/66 Dementia Research Group have devised, and extensively validated, a battery of assessments designed for use in the developing world and low literacy populations. These are intended to address some of the difficulties with dementia diagnosis in low- and middle-income countries (6). A cross-sectional study of dementia prevalence across 11 sites in 7 low- and middle-income countries by the 10/66 Research Group found the age-standardised prevalence of dementia, as defined by the 10/66 protocol (‘10/66 dementia’), to vary between 4.8% in rural China and 12.6% in Cuba (5). The prevalence of dementia according to the DSM-IV, calculated using a computerised algorithm, was found to be consistently lower than that of ‘10/66 dementia’, varying between 6.3% in Cuba and 0.3% in rural India. The authors suggest that the DSM-IV criteria may consistently underestimate the true prevalence of dementia by only including more severe cases. They argue that the 10/66 criteria help to identify cases of recent onset dementia and of mild cognitive impairment.

We have previously reported the results of a dementia prevalence study in people aged 70 and older, conducted in the rural Hai district of northern Tanzania (7). We found the prevalence of dementia to be 6.4% (95% CI 4.9–7.9) according to the DSM-IV criteria. The aim of the study was to compare the prevalence of dementia in Hai using both the DSM-IV and the 10/66 diagnostic criteria and to identify which criteria may represent the most appropriate method for identifying people with dementia in SSA.

## Methods

The National Institute of Medical Research, Dar-es-Salaam, Tanzania, approved this study.

### Study population

Tanzania is one of the world's poorest countries, with a gross national income per capita of $540 in 2011 (8). The Hai district in the north of the country is part of the Kilimanjaro region and is largely rural. The economy is based around agriculture with most people reliant on smallholdings as a source of income and food. On larger farms, cash crops, such as coffee, are grown providing a valuable additional source of income for many families. Part of Hai has been used as a demographic surveillance site (DSS) since the 1990s (9). As such, there are regular population censuses within the Hai DSS. On 1 June 2009, the population of all 52 villages of the Hai DSS was 161,119. We aimed to interview and assess all those aged 70 and older living in each of six villages, selected by a random number generator. Full details of the study recruitment process have been published previously (7).

### Assessment for the presence of dementia

#### Phase I

A census enumerator, who was trained in dementia screening by members of the study team, visited subjects who met the study inclusion criteria. Prior to commencement of the study, workshops were held for all enumerators and the concept of dementia was discussed at length. The enumerators were highly experienced in conducting community-based studies in Hai (10). Screening was conducted using the Community Screening Instrument for Dementia (CSI-D), which has been used extensively in low- and middle-income countries (11). The CSI-D has also been validated in Swahili, in a study from Kenya (12). The screening interview has two sections, with an interview for the person suspected of having dementia and an informant section for which a close family member is interviewed. The interview takes around 30–40 min to administer. These data are then fed into a computerised algorithm that classifies subjects as ‘probable dementia’, ‘possible dementia’ or ‘no dementia’ depending on their score (11).

#### Phase II

For all cases identified as having probable dementia, a full 10/66 interview protocol was administered by a member of the study team (S-MP, AL, AK) (13). We also aimed to administer the full 10/66 protocol to 50% of possible dementia cases and 5% of non-demented cases, as defined by the CSI-D. Possible and no dementia cases were selected for assessment using a random number generator.

### Dementia diagnosis

#### We report two different measures of dementia prevalence

##### 10/66 Dementia

The 10/66 Dementia Research Group has developed a battery of cognitive tests designed to allow a diagnosis of dementia to be made. The battery consists of four elements; the CSI-D cognitive and informant sections, the Geriatric Mental State (GMS) examination, and the Consortium to Establish a Registry for Alzheimer's Disease (CERAD) 10 word task. For those assessed during phase II, a diagnosis of ‘10/66 dementia’ was calculated using a computerised algorithm (5, 13). A prevalence rate for the entire denominator population was extrapolated from these phase II data based on the percentage of those with probable, possible, and no CSI-D dementia who were identified as having ‘10/66 dementia’.

##### Clinically diagnosed DSM-IV dementia

This was based on clinical assessment by a UK-trained doctor specialising in elderly care or psychiatry, with an interest in dementia (AL, S-MP). Diagnoses were checked by a UK-based Consultant Old Age Psychiatrist with an interest in dementia, who had access to clinical notes, during face-to-face discussion after the fieldwork was completed.

### Statistical methods

The methods for the calculation of dementia prevalence have been described previously (7). They are described here briefly. The denominator population was defined as those aged 70 and older who were resident in the six surveillance villages on the prevalence date, 12 April 2010. The numerator for the study was calculated based on all cases of dementia identified by the study team between 12 April and 30 September 2010 in these villages. Prevalence figures were calculated by weighting each case according to their initial CSI-D diagnosis.

Age standardisation, using the direct method, was to the WHO world standard (14). Confidence intervals (CIs) for prevalence were calculated using jackknife methods and adjusted for clustering of subjects by village (15). Logistic regression models were constructed to investigate the independent influence of formal education level on dementia prevalence having adjusted for the effect of age (stratified by age band) and gender. CIs for odds ratios (ORs) were calculated using the assumptions of the binomial distribution. The models were constructed using forced entry. The results are based on responses obtained during phase II.

## Results

The six villages had a population of 1,260 people aged 70 and older on the prevalence date. A total of 62 people refused to participate in the study and so the CSI-D was administered to 1,198 people, of whom 673 (56.2%) were female. From CSI-D screening in phase I, 184 people (15.4%) had ‘probable dementia’, of whom 125 (67.9%) were female. A further 104 people (8.7%) had ‘possible dementia’, of whom 68 (65.4%) were female.

A total of 168 people with ‘probable dementia’ according to the CSI-D were followed up during phase II. Sixteen people with ‘probable dementia’ could not be followed up due to death having occurred (*n*=8), having moved away (*n*=6) or being otherwise untraceable (*n*=2). In accordance with the study protocol, 56 (53.8%) people with ‘possible dementia’ and 72 (7.9%) people with ‘no dementia’ were also followed up. Cases of ‘10/66 dementia’ and clinically diagnosed DSM-IV dementia are shown in Table 1. From these data, extrapolated dementia rates were calculated. We have previously reported that the age standardised prevalence of DSM-IV dementia was 6.4% (95% CI 4.9–7.9) (7). The age standardised prevalence of dementia according to the 10/66 criteria was significantly higher, at 21.6% (95% CI 17.5–25.7). Of 78 cases with DSM-IV dementia, 75 (96.2%) were also diagnosed with ‘10/66 dementia’. These figures relating to the extent of agreement are similar to those of Llibre Rodriguez et al. (5).

**Table 1:** The prevalence of 10/66 and clinically diagnosed DSM-IV dementia

	Cases within 168 people with ‘probable dementia’	Cases within 56 people with ‘possible dementia’	Cases within 72 people with ‘no dementia’	Extrapolated dementia prevalence (%)
10/66 dementia				
Females	103	37	2	28.5
Males	45	7	1	16.3
70–74 years	22	5	1	13.1
75–79 years	33	8	1	19.4
80–84 years	27	13	1	29.7
≥ 85 years	66	18	0	43.7
Total all cases	148	44	3	23.5
Age-standardised all cases (14)	–	–	–	21.6
DSM-IV clinically diagnosed dementia				
Females	51	5	0	9.3
Males	22	0	0	4.8
70–74 years	9	1	0	3.5
75–79 years	10	1	0	3.8
80–84 years	15	1	0	8.6
≥ 85 years	39	2	0	19.3
Total all cases	73	5	0	7.5
Age-standardised all cases (14)	–	–	–	6.4

### Education

Educational data were available for 1,186 people (99.0%). Of 668 females, 419 (62.7%) had no education at all and 205 (30.7%) had 4 years or less of education; only 44 (6.8%) had more than 4 years of education. In comparison, of 518 males 166 (32.0%) had no education at all, 256 (49.4%) had 4 years of education or less and 96 (18.5%) had more than 4 years of education. Females were 3.56 times (95% CI 2.80–4.55) more likely to have had no education than males.

Regression models were constructed to investigate the influence of education on diagnosis within people assessed during phase II (Table 2). After adjusting for the effects of age and gender, people with no formal education were significantly more likely to be diagnosed with dementia according to the 10/66 criteria than people who had at least a period of primary school education (OR 2.225, 95% CI 1.284–3.855). However, having had no formal education was not an independent predictor of DSM-IV diagnosis (OR 1.108, 95% CI 0.619–1.985).

**Table 2:** Logistic regression models of the role of education after adjusting for the effect of age and gender

				95% CI for OR	
				
	B	Sig.	OR	Lower	Upper
10/66 dementia					
70–74 years	–	–	1	–	–
75–79 years	0.894	0.016	2.444	1.180	5.061
80–84 years	1.406	0.001	4.079	1.832	9.082
85 years or over	1.817	<0.001	6.156	2.938	12.898
Female gender	0.633	0.030	1.884	1.065	3.333
Education*	0.800	0.004	2.225	1.284	3.855
Constant	−0.837	0.005	0.433		
Clinical DSM-IV dementia					
70–74 years	–	–	1	–	–
75–79 years	0.147	0.759	1.158	0.454	2.952
80–84 years	0.807	0.077	2.242	0.918	5.478
85 years or over	1.279	0.002	3.592	1.619	7.971
Female gender	0.230	0.456	1.259	0.688	2.304
Education*	0.103	0.730	1.108	0.619	1.985
Constant	−1.940	<0.001	0.144		

OR = odds ratio, CI = confidence interval.

* Education was coded as 1 = none, 0 = some primary education or higher level. There were 13 missing values and the model is based on 283 cases.

### Other diagnoses

Of the 78 cases with clinically diagnosed DSM-IV dementia, 3 (3.8%) had a secondary diagnosis of depression, whilst of 96 people who had ‘probable dementia’ by CSI-D, but did not have clinical DSM-IV dementia, 26 (27.1%) had a primary or secondary diagnosis of depression and 50 (52.1%) were given an alternative psychiatric diagnosis, such as learning disability, schizophrenia or severe depression.

## Discussion

### Reasons for the differences in reported prevalence

Prevalence rates reported using the 10/66 diagnostic criteria were 3.38 times higher than when using the DSM-IV criteria. These results are in line with those reported by Llibre Rodriguez et al. (5). They found the rates of ‘10/66 dementia’ in Peru, China, Cuba, Venezuela, and Mexico to be 2–3 times higher than found using the DSM-IV criteria. There may be a number of reasons for this large discrepancy within our population. During data collection for the 10/66 protocol, case and informant fatigue with questioning was perceived to be a problem on occasion, although this was generally during phase I, where enumerators carried out the screening interview. Also, a number of subjects identified as having CSI-D probable dementia were subsequently given an alternative psychiatric diagnosis following clinical assessment in phase II. In our experience, another source of inflation of dementia prevalence rates when using diagnostic tests is sensory impairment, particularly eyesight impairment and hearing difficulty, which may negatively impact on the ability of a person to perform a task (e.g. the 10-word delayed recall test (hearing) or tests involving drawing (vision)). In the current study, 64.1% of those seen in phase II reported some form of eyesight impairment and 28.2% reported some form of hearing problem; such problems were almost always uncorrected. Allowing for a full medical (doctor-led) examination, as in the current study, identifies these physical impairments easily and clarifies the diagnosis, whereas relying solely on a lay person interview or use of a 10-word learning list only, may miss this and lead to false positives.

Other possible reasons for the large discrepancy in prevalence estimates have been discussed previously (5, 7). The DSM-IV criteria rely heavily on functional and occupational capacity and levels of social engagement. The lives of many older people living in rural SSA are often less cognitively demanding than those of their peers in high-income countries. As a consequence, it is possible that some people who would be diagnosed with early or mild DSM-IV dementia in high-income countries would not be diagnosed at all in SSA. Furthermore, many elderly cognitively impaired people in SSA have few functional and social limitations due to the protective nature of the extended family unit. It is generally acknowledged that management and treatment of dementia is most effective if done during the early stages of the disease (1). Therefore, use of the 10/66 criteria may be more appropriate when identification of people with mild cognitive impairment for management and treatment intervention is required. The 10/66 Dementia Research Group argue that the DSM-IV criteria may be too strict and may miss recent onset dementia and cases of mild cognitive impairment, which may go on to develop into dementia. There is undoubtedly much evidence to support this view. Nevertheless, it must be acknowledged that there may be little to be gained in diagnosing someone with dementia if they, their relatives and carers, do not identify any significant impairment in social or occupational capacity. Within our study we were particularly mindful of the need for such impairment to be identified, within accepted cultural norms for our study population. We employed an experienced nurse from the local area to avoid misinterpretation in this regard. The development and validation of a scale to measure impairment in functional activities of daily living specific to SSA should be considered. Such a scale could be used to inform a DSM-IV dementia diagnosis.

Neurological and psychiatric conditions, including dementia, are often poorly understood by the general population in SSA, and may be attributed to witchcraft or evil spirits, rather than identifiable medical conditions (16). This can result in significant stigma associated with such diseases. Such stigma has been suggested as a reason for relatively low prevalence rates for dementia in low- and middle-income countries (17). Cases are sometimes hidden and there is a reluctance to seek medical help. This problem is exacerbated by the fact that dementia symptoms are often seen as a normal part of the ageing process, rather than a specific medical condition (18). Our use of village enumerators to explain the study and allay fears regarding stigma for families and carers helped to avoid such biases and emphasises the advantage of involving local research staff during epidemiological studies. This is especially true when cultural issues surrounding a disease may make it a sensitive topic for discussion. It has been noted that the CSI-D, a major element of the 10/66 diagnosis, may classify some cases of depression as dementia (13). Our results would tend to support this.

Although the 10/66 diagnosis may include cases of mild cognitive impairment, it is difficult to argue that these should be classified as dementia cases for the purposes of a prevalence study. Nevertheless, identification of people who may go on to develop dementia is likely to be of benefit to clinicians and healthcare managers working in resource-poor settings, such as SSA.

### The effects of age and gender

As expected, dementia prevalence increased with increasing age using both diagnostic criteria and was significantly more common in older age groups, even after adjusting for the effects of gender and education (Table 2). Dementia was also more common in females than males, however this was not significant when using the DSM-IV criteria after adjusting for the effects of age and education; women tended to be older and have less formal education. Using the 10/66 criteria, females were more likely to have dementia than males, even after adjusting for other effects. It is not clear why females should be more likely to have dementia than males using the 10/66 criteria, but not the DSM-IV criteria. The smaller number of cases identified by the DSM-IV criteria, limiting the power of any statistical test, may be one of the main reasons. However, it is also possible that females, having a higher average age, may have had greater risk of co-morbidity (such as hearing loss, visual impairment, and physical impairment) and therefore more likely to be diagnosed with dementia according to the 10/66 criteria. The effect of such impairments may not have been fully accounted for by adjusting for the effects of age or education alone.

### The role of education

In our study, there was no significant difference in clinically diagnosed DSM-IV dementia rates with education level after adjusting for the effects of age and gender. There was, however, a significant difference in 10/66 dementia rates with education level after adjusting for these factors. Whether the differences in prevalence with education level reflect an educational bias in the 10/66 diagnostic criteria when used in SSA or a genuine effect is not clear. In our study, certain parts of the 10/66 battery of tests were generally poorly performed. Most notably the elements of the CSI-D that require the interviewee to draw various shapes with a pencil. Since many people in our population had never held a pen or pencil before, coupled with a high prevalence of visual impairment, it was felt that poor performance may not have been entirely due to cognitive impairment.

Conversely, it may be that the influence of education level is confounded with other variables, such as socio-economic status or childhood morbidity, which may play a part in the onset of dementia. This has been noted in a recent study in urban Brazil (19). Furthermore, as Guerchet et al. (20) point out, in SSA the formal schooling level is often a poor marker for education level, since senior family members teach many children informally. Again, the smaller number of cases identified using the DSM-IV criteria may be another reason for the lack of significance for any differences in DSM-IV dementia rates with education. However, the adjusted OR for education using the DSM-IV criteria is close to unity (1.108), compared to the much higher adjusted OR using the 10/66 criteria (2.225), suggesting that this may not be the primary reason.

### Limitations

Single-phase studies may be an ideal for any prevalence study (6, 21). However, two-phase studies are a more pragmatic approach to research when carrying out a full clinical assessment of large populations is not possible due to resource limitations. Phase II was carried out within 6 months of phase I screening, and we were able to fully assess 91.3% of probable dementia cases identified during phase I. The most common reason for loss to follow-up was death; occurring in 50% (*n*=8) of those we were unable to follow-up. These attrition rates are slightly lower than those reported by Guerchet et al. (22) and emphasise the practicality of two-phase studies. Although it is possible that those not followed up due to death may have been more likely to have dementia than those who were followed up, the numbers involved were relatively small and we do not feel our results will have been significantly biased.

## Conclusions

Although other methods of diagnosing dementia exist, the clinical DSM-IV criteria allow direct comparison between populations in different world regions. Since it is a clinical diagnosis, with an emphasis on limitations of functional capacity caused by dementia, it reflects the effect of the disease on the individual. Furthermore, the clinical nature of the criteria may make the diagnosis less susceptible to educational bias. People that fall under the DSM-IV criteria are likely to be the most recognisable as having dementia as it would be defined in high-income countries. Therefore, for the purposes of documenting prevalence, we feel the DSM-IV criteria should be used, though we recognise that in developing world settings it may represent an under-estimate.
